# Extracellular MRP8/14 is a regulator of β2 integrin-dependent neutrophil slow rolling and adhesion

**DOI:** 10.1038/ncomms7915

**Published:** 2015-04-20

**Authors:** Monika Pruenster, Angela R. M. Kurz, Kyoung-Jin Chung, Xiao Cao-Ehlker, Stephanie Bieber, Claudia F. Nussbaum, Susanne Bierschenk, Tanja K. Eggersmann, Ina Rohwedder, Kristina Heinig, Roland Immler, Markus Moser, Uwe Koedel, Sandra Gran, Rodger P. McEver, Dietmar Vestweber, Admar Verschoor, Tomas Leanderson, Triantafyllos Chavakis, Johannes Roth, Thomas Vogl, Markus Sperandio

**Affiliations:** 1 Institute of Cardiovascular Physiology and Pathophysiology, Walter-Brendel-Centre of Experimental Medicine, Ludwig-Maximilians Universität, Munich, Germany; 2 Department of Clinical Pathobiochemistry, Institute for Clinical Chemistry and Laboratory Medicine, Technische Universität Dresden, Dresden, Germany; 3 Department of Molecular Medicine, Max Planck Institute for Biochemistry, Martinsried, Germany; 4 Department of Neuroinflammation, Ludwig-Maximilians Universität, Munich, Germany; 5 Institute of Immunology, University of Muenster, Muenster, Germany; 6 Cardiovascular Biology Research Program, Oklahoma Medical Research Foundation, Oklahoma City, OK, USA; 7 Max Planck Institute for Biomolecular Medicine, Münster, Germany; 8 Institute for Medical Microbiology, Immunology and Hygiene, Technische Universität, Munich, Germany; 9 Immunology Group, University of Lund, Lund, Sweden

## Abstract

Myeloid-related proteins (MRPs) 8 and 14 are cytosolic proteins secreted from myeloid cells as proinflammatory mediators. Currently, the functional role of circulating extracellular MRP8/14 is unclear. Our present study identifies extracellular MRP8/14 as an autocrine player in the leukocyte adhesion cascade. We show that E-selectin–PSGL-1 interaction during neutrophil rolling triggers Mrp8/14 secretion. Released MRP8/14 in turn activates a TLR4-mediated, Rap1-GTPase-dependent pathway of rapid β2 integrin activation in neutrophils. This extracellular activation loop reduces leukocyte rolling velocity and stimulates adhesion. Thus, we identify Mrp8/14 and TLR4 as important modulators of the leukocyte recruitment cascade during inflammation *in vivo.*

## Abstract (web summary)

MRP8/14 are actively secreted by myeloid cells during inflammation. Here the authors show that MRP8/14 play an important role in leukocyte recruitment to the inflammatory site, triggering an autocrine cascade that promotes neutrophil adhesion to the endothelium.

## Graphical abstract

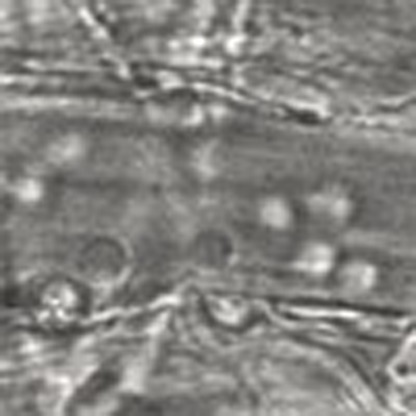

Myeloid-related protein 8 (MRP8, also known as S100A8 or calgranulin A) and myeloid-related protein 14 (MRP14, S100A9, calgranulin B) are two members of the S100 family, characterized by two calcium-binding EF-hand motifs, which are connected by a central hinge region^1^,^2^. They form a heterocomplex (MRP8/14, also known as calprotectin), which is considered to be the physiologically relevant form^3^, and are found in the cytosol of neutrophils representing 40% of the cytosolic protein content in neutrophils^4^. The Mrp8/14 complex is released from myeloid cells during inflammatory events through an alternative, Golgi-independent route^1^,^2^ and exerts its proinflammatory effect on endothelial cells^5^, phagocytes^6^,^7^,^8^ and lymphocytes^9^. Recently, it has been shown that Mrp8/14 may also be involved in the regulation of adaptive immune responses^10^. Several inflammatory disorders including chronic bronchitis^11^, inflammatory bowel disease^12^,^13^, rheumatoid arthritis^14^, psoriasis^15^ or systemic-onset juvenile idiopathic arthritis^16^ are associated with elevated serum levels of MRP8/14. As a consequence, these proteins are suitable biomarkers of inflammation and are used to monitor the response towards anti-inflammatory therapy^14^,^17^,^18^. Interestingly, the exact function of serum-circulating MRP8/14 during an inflammatory response has remained elusive. This has also been attributed to the fact that the intracellular function of MRP8/14 may be fundamentally different to its extracellular function as an alarmin molecule^1^.

In inflammation, leukocyte recruitment follows a defined cascade of adhesion and activation events beginning with selectin-mediated leukocyte capture to and rolling along the inflamed endothelium^19^,^20^. This triggers intermediate activation of β2 integrins and consecutive slowing down of the rolling leukocytes. Furthermore, rolling facilitates binding of leukocyte-expressed chemokine receptors to their cognate chemokines presented on the luminal endothelial surface. This in turn induces, together with E-selectin, binding to P-selectin glycoprotein-1 (PSGL1) and full activation of β2 integrins^21^,^22^ leading to firm leukocyte adhesion to the endothelial surface.

Extracellular MRP8/14 is thought to influence different steps along the leukocyte recruitment process via binding to surface receptors including the Toll-like receptor-4 (TLR4) and the receptor of advanced glycation endproducts (RAGE). It has been shown that extracellular MRP8/14 induces endothelial secretion of the inflammatory chemokine CXCL8 (CXC chemokine ligand 8 also known as IL8) and upregulates Intercellular Adhesion Molecule 1 (ICAM-1) on human microvascular endothelial cells (HMECs) *in vitro*^5^. In addition, cellular permeability of MRP8/14-stimulated-HMEC monolayers increased^5^. Concerning the effect of MRP8 and MRP14 on leukocyte adhesion, Newton and Hogg showed that human MRP14 stimulates neutrophil adhesion to fibrinogen by activating the β2 integrin Mac-1 (ref. 7). Despite recent progress regarding the function of extracellular MRP8 and MRP14 on leukocyte recruitment, the molecular mechanisms triggering the release of MRP8/14 into the extracellular compartment and the functional outcomes of such a release have remained elusive. In our study, which focused on the extracellular function of MRP8/14, we show that neutrophil rolling on E-selectin present on inflamed endothelium leads to Mrp8/14 secretion. Extracellular Mrp8/14 in turn acts in an autocrine manner through an extracellular activation loop via binding to its receptor TLR4 on neutrophils, which subsequently induces the activation of Rap1 and β2 integrins and neutrophil slow rolling and firm arrest. Thus, we present a novel mechanism of rapid TLR4-induced β2 integrin activation involving extracellular MRP8/14 as a modulator of leukocyte recruitment that bridges the steps of rolling and adhesion during an inflammatory response *in vivo*.

## Results

### E-selectin triggers Mrp8/14 release *in vitro* and *in vivo*

**Figure 1:**
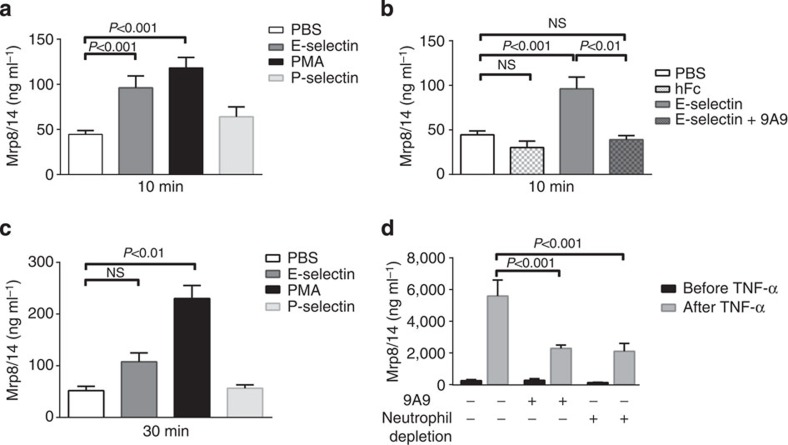
Interaction of neutrophils with E-selectin results in Mrp8/14 release *in vitro* and *in vivo.* Bone marrow-derived neutrophils from C57BL/6 WT mice were incubated at 37 °C in wells pre-coated with E-selectin, P-selectin or PBS. Soluble PMA was used as positive control. Supernatants were collected and analysed by ELISA. Mrp8/14 secretion (mean±s.e.m., *n*≥5 mice per group, one-way analysis of variance (ANOVA) with Dunnett's *post-hoc* test) induced by E-selectin (grey bar), PMA (black bar) and P-selectin (light grey bar) is shown after 10 min (**a**) and 30 min (**c**) incubation. (**b**) Mrp8/14 secretion (mean±s.e.m., *n*≥3 mice per group, one-way ANOVA with Dunnett's *post-hoc* test) after 10 min induced by human Fc control (white spotted bar), E-selectin (grey bar, same bar as in **a**) and E-selectin+anti E-selectin antibody 9A9 (grey spotted bar). PBS was used as control (white bar, same bar as in **a**). (**d**) *In vivo* Mrp8/14 secretion induced by E-selectin: rmTNF-α was injected into the scrotum of C57BL/6 WT mice without or with E-selectin blockade (9A9) and after neutrophil depletion (*n*≥3 mice per group, two-way ANOVA with Tukey's *post-hoc* test).

Recent studies showed that MRP8/14 was released during the interaction of activated monocytes with tumor-necrosis factor (TNF)-α-stimulated, but not resting human umbilical vein endothelial cells (HUVECs)^16^. Here, we investigated which signals on the inflamed endothelium were able to induce MRP8/14 release from activated phagocytes. To this end, we isolated bone marrow-derived neutrophils from C57BL/6 wild-type (WT) mice and incubated the cells at 37 °C in wells pre-coated with E-selectin-Fc, P-selectin-Fc or PBS. Soluble phorbol myristate acetate (PMA) was used as a positive control. Supernatants were collected 10 and 30 min thereafter and analysed for Mrp8/14 by ELISA as described previously^23^. Incubation (10 min) of isolated neutrophils with PBS induced Mrp8/14 secretion (42±4 ng ml^−1^; Fig. 1a). Secretion was significantly increased in the presence of E-selectin (96±13 ng ml^−1^) and PMA (118±12 ng ml^−1^). In contrast, the presence of P-selectin failed to induce a significant increase in Mrp8/14 release (64±11 ng ml^−1^) in this assay. Addition of rat anti-mouse E-selectin antibody 9A9 inhibited E-selectin-induced Mrp8/14 release (39±5 ng ml^−1^; Fig. 1b). Fc control was unable to induce Mrp8/14 release (30±7 ng ml^−1^). Similar results were found after 30 min incubation (PBS: 52±8 ng ml^−1^, E-selectin: 108±17 ng ml^−1^ and P-selectin: 56±7 ng ml^−1^) with the exception of PMA, which further increased the release of Mrp8/14 (230±25 ng ml^−1^; Fig. 1c).

To evaluate E-selectin involvement in Mrp8/14 secretion under *in vivo* conditions, we injected recombinant mouse TNF-α (rmTNF-α) into the scrotum of C57BL/6 WT mice with or without intravenous (i.v.) injection of E-selectin-blocking monoclonal antibody 9A9 (30 μg per mouse) 15 min before rmTNF-α application. Mrp8/14 serum levels were measured before and 2 h after rmTNF-α treatment by ELISA. Mrp8/14 serum levels of untreated mice were 249±66 and 273±105 ng ml^−1^, respectively. Application of rmTNF-α increased serum levels of Mrp8/14 to 5,590±1,013 ng ml^−1^. Pretreatment of mice with anti E-selectin blocking monoclonal antibody 9A9 significantly reduced serum levels of Mrp8/14 to 2,292±206 ng ml^−1^, indicating that E-selectin is involved in Mrp8/14 release *in vivo* (Fig. 1d). In a second set of experiments, we depleted neutrophils from mouse blood to prove that serum Mrp8/14 is neutrophil-derived in this *in vivo* model. For this approach, we pretreated mice with anti-Ly6C antibody 1A8 (30 μg per mouse) 24 h before rmTNF-α treatment. Mrp8/14 serum levels of neutrophil-depleted mice were 130±19 ng ml^−1^ before rmTNF-α treatment and 2,100±505 ng ml^−1^ after rmTNF-α treatment (Fig. 1d). These findings highlight that neutrophils are a central source of serum circulating Mrp8/14 under inflammatory conditions.

### E-selectin-triggered Mrp8/14 release is PSGL1 dependent

**Figure 2:**
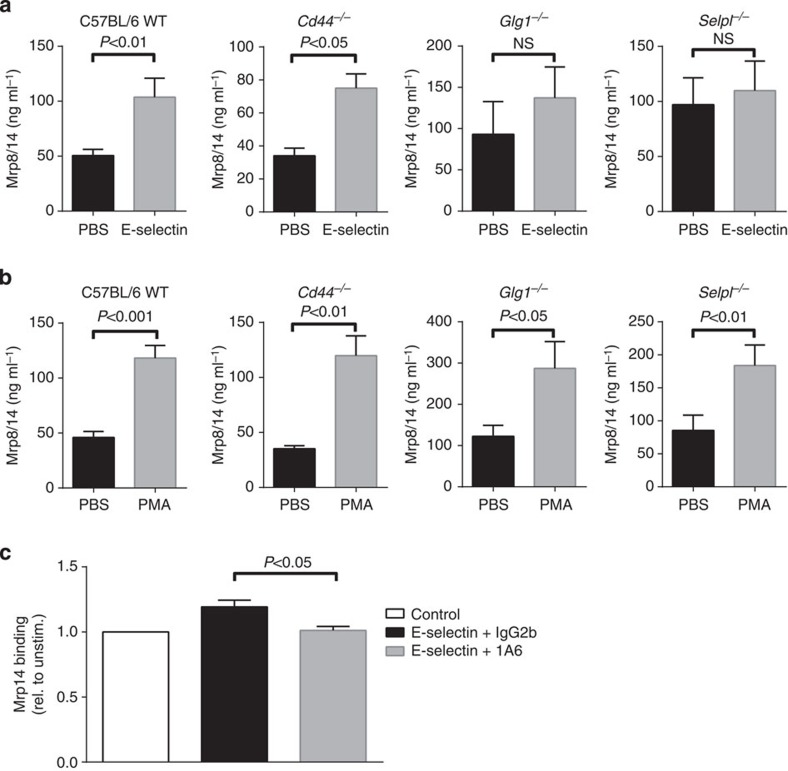
E-selectin-triggered Mrp8/14 release is PSGL1-dependent and -independent of CD44. Bone marrow neutrophils were isolated from C57Bl/6 WT, *Selpl*^*−/−*^, *Cd44*^*−/−*^ and *Glg1*^*−/−*^ mice and incubated on wells coated with PBS or E-selectin (**a**) or incubated with PMA (**b**) for 10 min. Supernatants were collected and Mrp8/14 release was determined by ELISA (*n*≥3 mice per group, paired t-Test). Data are presented as mean±s.e.m. In addition, Mrp8/14 binding to TLR4 on leukocytes was assessed (**c**). Bone marrow cells from C57BL/6 WT mice were isolated and stimulated for 5 min at 37 °C with or without soluble rmE-Selectin. The amount of extracellular surface-bound Mrp14 protein on Ly6G+/Mrp14+ leukocytes was determined by flow cytometry. Mean fluorescence intensity values of cells stimulated without E-Selectin (control) were set to 1 (white bar) and values of E-selectin-stimulated cells were calculated as mean ratio compared with control. Data are presented as mean±s.e.m. (*n*=4 mice per group, unpaired *t*-test).

Under *in vivo* conditions, E-selectin is known to bind to three different ligands on mouse neutrophils: PSGL1 (gene name: *Selpl*), CD44 and ESL1 (gene name: *Glg1*)^24^. To identify the E-selectin ligand responsible for E-selectin-triggered Mrp8/14 release, we isolated bone marrow neutrophils from C57Bl/6 WT, *Selpl*^*−/−*^ and *Cd44*^*−/−*^ mice, as well as from mice with a *Glg1*-deficient haematopoietic system. We incubated the cells on wells coated with PBS or E-selectin for 10 min, collected the supernatants and analysed the samples for Mrp8/14 release by ELISA as described^23^ (Fig. 2a). PMA was used as a positive control (Fig. 2b). Lack of CD44 did not influence the capability of cells to secrete Mrp8/14. *Cd44*^*−/−*^ cells incubated with PBS secreted 35±3 ng ml^−1^ Mrp8/14. Incubation of the cells with E-selectin increased the levels to 75±9 ng ml^−1^ (Fig. 2a). Cells from *Glg1*^*−/−*^- and *Selpl*^*−/−*^-deficient mice exhibited an increased basal Mrp8/14 release as compared with C57BL/6 WT or *Cd44*^*−/−*^ cells (93±40 ng ml^−1^ and 97±25, respectively). *Glg1*^*−/−*^-deficient cells stimulated with E-selectin showed a slight, however, not significant, increase in Mrp8/14 secretion (137±37 ng ml^−1^). *Selpl*^*−/−*^ cells incubated on E-selectin-coated plates were unable to increase Mrp8/14 levels as compared with levels induced by PBS (110±27 ng ml^−1^). These results suggest an involvement of PSGL-1 in E-selectin-triggered Mrp8/14 release. Whether ESL1 may have an additional role in E-selectin-dependent secretion of Mrp8/14 is not entirely clear and needs further investigation. Secretion of Mrp8/14 upon PMA stimulation was not affected in *Cd44*^*−/−*^-, *Glg1*^*−/−*^- and *Selpl*^*−/−*^-deficient cells (120±18, 288±65 and 184±31 ng ml^−1^, respectively, Fig. 2b).

### Released Mrp8/14 binds to TLR4 in an autocrine manner

Next, we tested the ability of released (soluble) Mrp8/14 to bind to leukocytes in an autocrine manner. For this approach, bone marrow cells from C57BL/6 WT mice were isolated and stimulated for 5 min at 37 °C with or without (control) soluble E-selectin. Mrp8/14 on neutrophils was detected using a polyclonal anti-mouse Mrp14 antibody, followed by a fluorescently labelled secondary antibody. As TLR4 is considered the main cellular surface receptor of Mrp8/14 on leukocytes^23^, experiments were performed in the absence or presence of rat anti-mouse TLR4 antibody 1A6 or IgG2b control. The amount of extracellular surface-bound Mrp14 protein on Ly6G^+^/Mrp14^+^ leukocytes was determined using FACS analysis. As shown in Fig. 2c, cells stimulated with E-selectin showed a 20% higher amount of Mrp14 protein (factor 1.20±0.04%) on the cellular surface compared with control cells. Addition of anti-mouse TLR4 antibody 1A6 completely abolished the ability of released Mrp14 protein to bind to neutrophils (factor 1.01±0.06), suggesting that released Mrp8/14 can bind to TLR4 in an autocrine manner. For representative FACS plots, see Supplementary Fig. 1.

### MRP8/14 activates β2 integrins via TLR4

**Figure 3:**
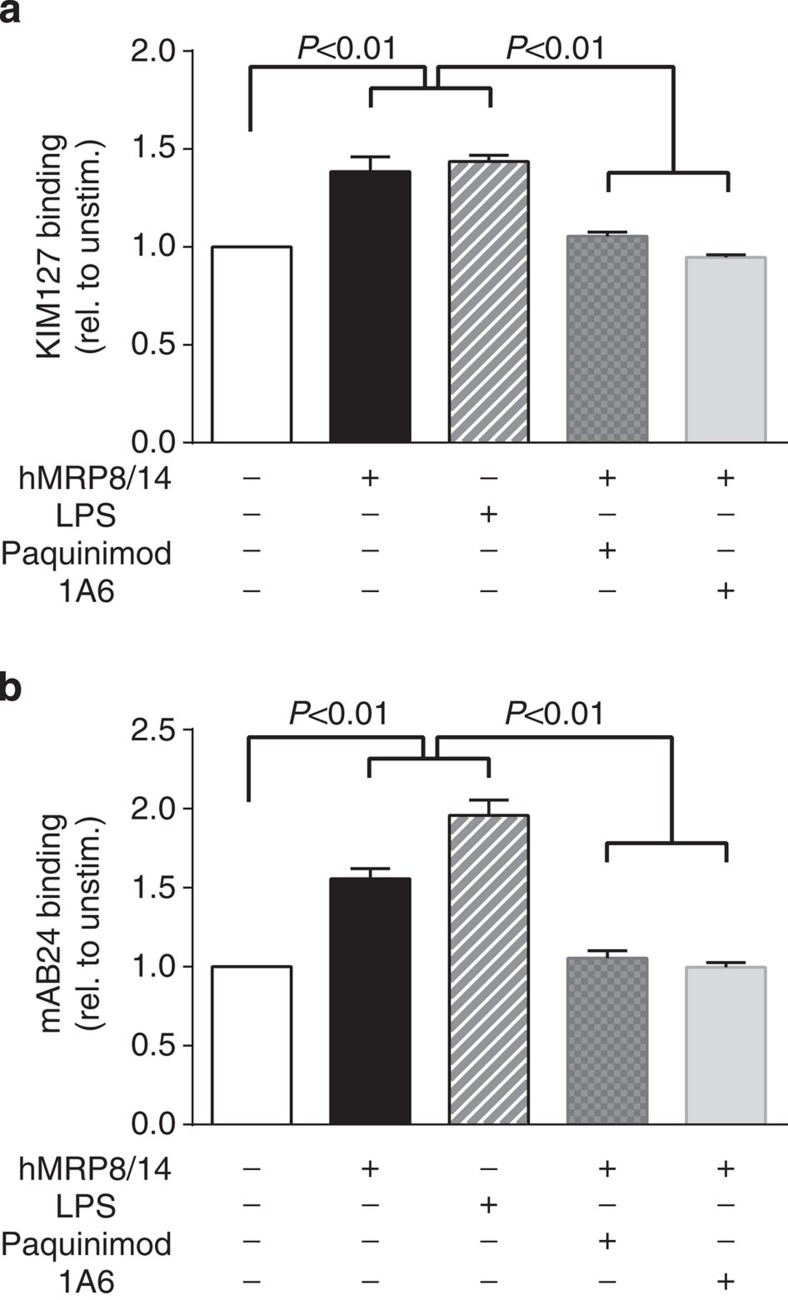
MRP8/14 activates β2 integrins via TLR4. Neutrophils from healthy blood donors were stimulated for 5 min at 37 °C with or without human MRP8/14 (hMRP8/14) or LPS. In some experiments, cells were preincubated with rat anti-human polyclonal TLR4 antibody or Paquinimod. Activation status of β2 integrins was determined using flow cytometry. Gates were set by using an isotype control. Values of KIM127 and mAB24 binding from unstimulated control was set to 1. (**a**) KIM127 binding and (**b**) mAb24 binding: control neutrophils (*n*=9, white bar), neutrophils incubated with hMRP8/14 (*n*=9, black bar), neutrophils incubated with LPS (*n*=3, white lined bar). Preincubation of hMRP8/14-stimulated cells with Paquinimod or polyclonal rat anti-human TLR4 antibody reduced KIM127 binding to control levels (*n*=6, grey spotted bar and *n*=3, grey bar, respectively). Data are presented as mean±s.e.m., one-way analysis of variance with Tukey's *post-hoc* test.

Next, we investigated the effects of the MRP8/14–TLR4 interaction on the activation status of β2 integrins. Owing to the lack of antibodies recognizing the activation status of β2 integrins in mice, we performed the following experiments using human blood neutrophils where respective activation-specific antibodies are available. We enriched neutrophils from healthy blood donors and stimulated the cells for 5 min at 37 °C with or without granulocytic human MRP8/14 (hMRP8/14) or lipopolysaccharide (LPS), another known TLR4 ligand. The activation status of β2 integrins was analysed by FACS using the β2 integrin activation markers KIM127 and mAB24 (ref. 25). Interestingly, both hMRP8/14 and LPS were able to convert β2 integrins into an activated form. Neutrophils incubated with hMRP8/14 showed 1.38±0.08-fold higher KIM127 antibody binding compared with control neutrophils (Fig. 3a). Incubation with LPS increased binding of KIM127 by a factor of 1.44±0.03. To prove that measured β2 integrin activation was indeed induced via hMRP8/14 interaction with TLR4, we pretreated human neutrophils with polyclonal rat anti-human TLR4 antibody or Paquinimod (ABR215757), respectively, before incubating the cells with hMRP8/14. Paquinimod is a small-molecule inhibitor, which blocks binding of MRP8/14 to its receptor TLR4 (refs 26, 27). Both Paquinimod and polyclonal rat anti-human TLR4 antibody abolished the activation of β2 integrins by hMRP8/14. Binding of mAB24 antibody was increased by a factor of 1.56±0.06 and 1.96±0.1 when cells were incubated with hMRP8/14 or LPS, respectively, as compared with control (Fig. 3b). Again, we were unable to measure β2 integrin activation by hMRP8/14 in the presence of Paquinimod or anti-human TLR4 antibody using activation-specific mAB24 antibody (factor 1.05±0.05 and 1.00±0.03, respectively). For representative histogramm plots, see Supplementary Fig. 2a,b. Surface expression levels of total LFA-1 and total Mac-1 were not influenced by hMRP8/14 or by LPS (Supplementary Fig. 3a,b).

### MRP8/14 activates Rap1 via TLR4

**Figure 4:**
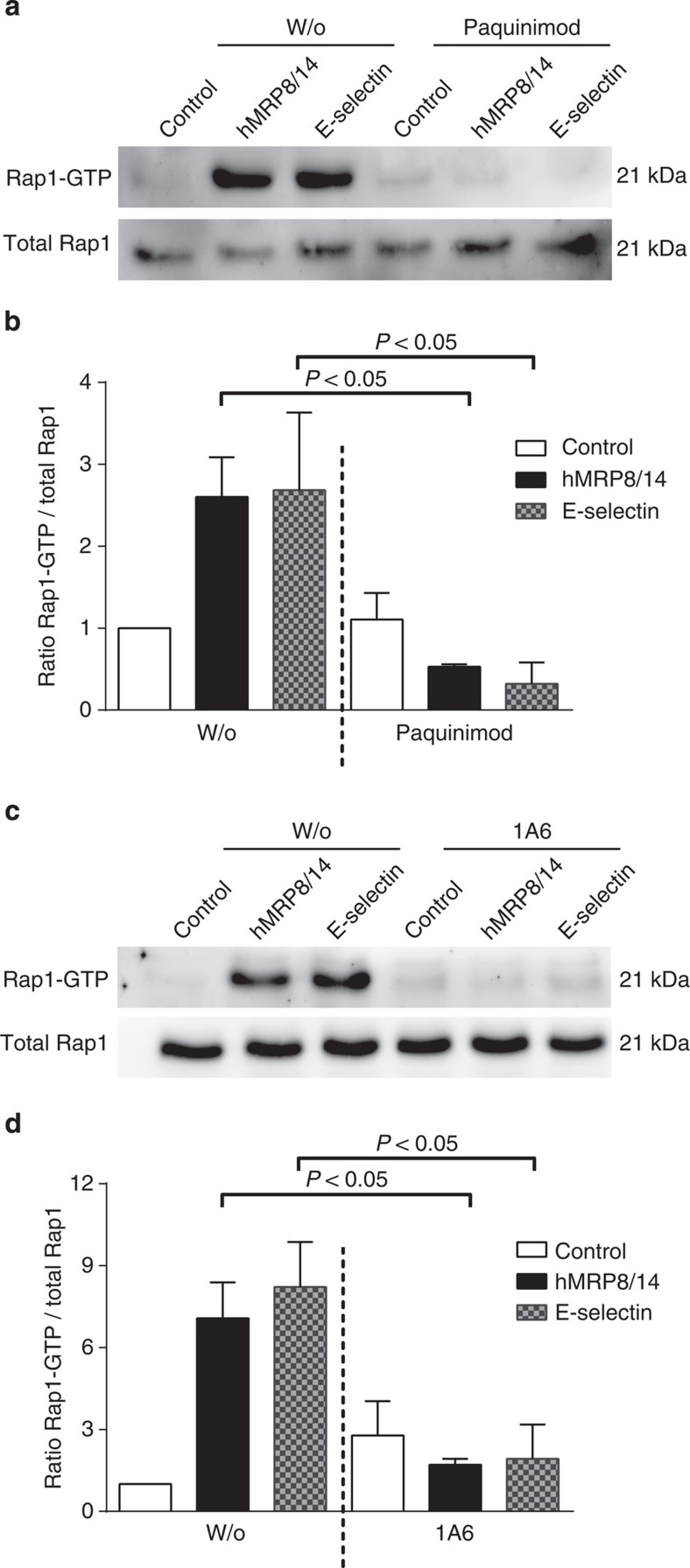
MRP8/14 activates Rap1. Representative immunoblots are shown for total Rap1 and Rap1-GTP from human neutrophils stimulated with PBS/0.1% BSA (control), human MRP8/14 (hMRP8/14) or recombinant human E-selectin (E-selectin) without (left panel, w/o) or with Paquinimod (right panel, Paquinimod; **a**) and without (left panel, w/o) or with rat anti-human TLR4 antibody 1A6 (right panel, 1A6; **c**). Density blots were calculated using Image J software (**b**,**d**). Data are presented as mean±s.e.m. (*n*=3 per group, one-way analysis of variance with Dunnett's *post-hoc* test).

The small GTPase Rap1 is involved as an intermediate of the E-selectin-triggered activation of β2 integrins and of the inside out signalling activation of integrins via G-protein-coupled receptors during leukocyte recruitment^28^. In its role as upstream molecule of inside-out signalling-mediated β2 integrin activation, it regulates binding affinity of β2 integrins and therefore rolling velocities and adhesive properties^28^. Here we wanted to test whether active Rap1 (GTP-Rap1) is also induced by hMRP8/14. For this approach, human neutrophils isolated from healthy blood donors were stimulated for 5 min at 37 °C in the presence or absence of hMRP8/14. Recombinant human E-selectin was used as positive control. Both hMRP8/14 and E-selectin induced an upregulation of Rap1-GTP in neutrophils (Fig. 4, left panels). Pretreatment of the cells with Paquinimod (Fig. 4a,b) or rat anti-human TLR4 antibody (Fig. 4c,d) abolished hMRP8/14- and E-selectin-induced Rap1 activation demonstrating that E-selectin, as well as hMRP8/14-triggered activation of Rap1 is dependent on TLR4. Full immunoblots are shown in Supplementary Fig. 4a,b. Heat inactivation of hMRP8/14 and E-selectin prevented Rap1 activation, excluding any relevant LPS contamination of proteins used in this assay (Supplementary Fig. 4b).

### Mrp8/14 reduces rolling velocity of neutrophils

**Figure 5:**
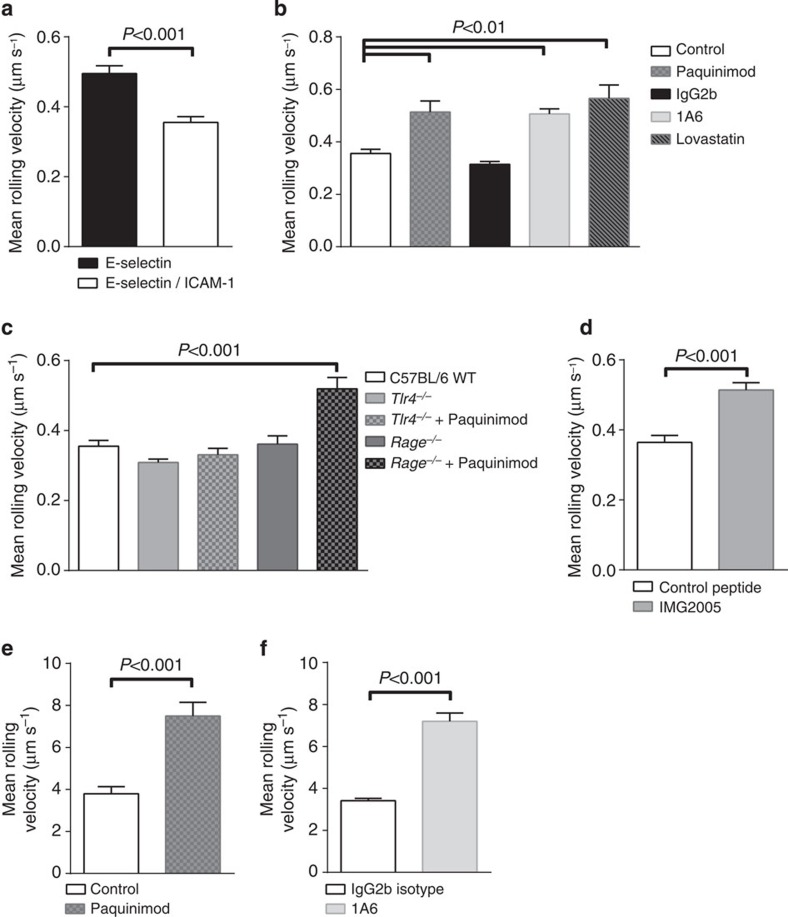
Mrp8/14 reduces rolling velocity of neutrophils *in vitro* and *in vivo.* Whole mouse blood was perfused through microflow chambers (shear stress 2.7 dyn cm^−2^, *n*≥3 mice per group). (**a**) Leukocytes rolling velocities of C57BL/6 WT mice were assessed in rmE-selectin (*n*=209 cells) and rmE-selectin/rmICAM-1-coated glass capillaries (*n*=178 cells). For rmE-selectin/rmICAM-1-coated glass capillaries, leukocyte rolling velocities were assessed for (**b**) untreated leukocytes (white bar, same bar as in **a**), Paquinimod-treated leukocytes (*n*=97 cells, grey spotted bar), rat IgG2b isotype-treated leukocytes (*n*=165 cells, black bar), anti-TLR4 antibody 1A6-treated leukocytes (*n*=184 cells, light grey bar) and Lovastatin-treated leukcoytes (*n*=65 cells, grey lined bar). In addition, leukocyte rolling velocities from C57BL/6 WT mice were assessed for (**c**) untreated leukocytes (white bar, same bar as in **a** and **b**), from *Tlr4*^*−/−*^ mice for untreated leukocytes (*n*=271 cells, grey bar) or Paquinimod treated leukocytes (*n*=182 cells, grey spotted bar) and from *Rage*^*−/−*^ mice for untreated leukocytes (*n*=88 cells, dark grey bar) or Paquinimod-treated leukocytes (*n*=140 cells, dark grey spotted bar). (**d**) *Ex vivo* leukocyte rolling velocities of cells treated with control peptide (*n*=140 cells, white bar) or MyD88 inhibitor IMG2005 (*n*=160 cells, grey bar) were assessed. (**e**) *In vivo* leukocyte rolling velocities were analysed in rmTNF-α-stimulated venules of mouse cremaster muscles of C57BL/6 WT mice, pretreated with PBS/10% DMSO as control (*n*=98 cells, white bar) or pretreated with Paquinimod (*n*=71 cells, grey spotted bar). (**f**) In addition, *in vivo* rolling velocity was assessed for mice pretreated with rat IgG2b isotype control (*n*=225 cells, white bar) or pretreated with rat anti-mouse TLR4 antibody 1A6 (*n*=214 cells, light grey bar). Data are presented as mean±s.e.m. (**a**,**d**,**e**,**f**) unpaired *t*-test, (**b**,**c**) one-way analysis of variance with Dunnett's *post-hoc* test.

As the activation status of β2 integrins directly influences leukocyte rolling velocity, we investigated the influence of secreted Mrp8/14 on leukocyte rolling behaviour. For this purpose, we used a slightly modified microflow chamber system^29^ coated with recombinant mouse E-selectin (rmE-selectin) alone or in combination with recombinant mouse ICAM-1 (rmICAM-1). The combination of rmE-selectin and rmICAM-1 mediates slow rolling of neutrophils and reflects the *in vivo* situation of cells rolling on TNF-α-stimulated postcapillary venules^21^. Whole mouse blood was obtained via a carotid artery catheter, heparinized and perfused through the microflow chamber using a high precision perfusion pump at a shear stress level of 2.7 dyn cm^−2^ (*n*≥3 mice per group). Leukocytes from untreated control blood rolled at a velocity of 0.50±0.02 μm s^−1^ on rmE-selectin-coated glass capillaries (Fig. 5a). A combination of rmE-selectin/rmICAM-1 reduced rolling velocity significantly to 0.36±0.02 μm s^−1^. This ICAM-1-dependent reduction is in accordance to previous findings^21^,^30^ and reflects the previously described E-selectin-induced intermediate activation of β2 integrins on leukocytes^21^. To test whether E-selectin-regulated rolling velocity depends on Mrp8/14 release and subsequent Mrp8/14 binding to TLR4, we investigated leukocyte rolling velocity on rmE-selectin/rmICAM-1-coated capillaries in the presence or absence of Paquinimod as well as in the presence or absence of rat anti-mouse TLR4 antibody 1A6. Addition of Paquinimod to the blood resulted in an increase in leukocyte rolling velocity to 0.51±0.04 μm s^−1^ (Fig. 5b). In line with this finding, the presence of anti-mouse TLR4 antibody 1A6 resulted in an increase in rolling velocity to 0.51±0.02 μm s^−1^. Rat IgG2b isotype control did not change the rolling velocities of cells rolling on rmE-selectin/rmICAM-1 when compared with untreated control cells (0.31±0.01 μm s^−1^). In addition, application of Lovastatin, which has been demonstrated to keep β2-integrins in their low-affinity status^31^, resulted in a mean rolling velocity of 0.57±0.05 μm s^−1^. Unexpectedly, whole blood cells from *Tlr4*-deficient mice (*Tlr4*^*−/−*^) did not show an increased rolling velocity (Fig. 5c). Cells from *Tlr4*^*−/−*^ mice rolled with a mean rolling velocity of 0.31±0.01 μm s^−1^. However, presence of Paquinimod did not influence rolling velocity of *Tlr4*^*−/−*^ cells (0.33±0.02 μm s^−1^), indicating that slow rolling is not induced via released Mrp8/14 in *Tlr4*^*−/−*^ cells, but due to another, yet undefined mechanism. To also test a potential contribution of another Mrp8/14 receptor, RAGE, for Mrp8/14-induced slow leukocyte rolling velocity, we used whole blood from *Rage*-deficient mice (*Rage*^*−/−*^). In contrast to *Tlr4*^*−/−*^ cells, we found leukocyte rolling velocities in *Rage*-deficient mice (0.52±0.03 μm s^−1^ with Paquinimod versus 0.36±0.02 μm s^−1^ without Paquinimod, Fig. 5c), which were comparable to WT cells. These findings indicate that RAGE is not involved as relevant receptor for Mrp8/14-dependent regulation of rolling velocity. Next, we investigated potential downstream signalling molecules involved in TLR4-dependent β2 integrin activation and slow rolling. For this approach, we pretreated C57BL/6 WT mice by intraperitoneal (i.p.) injection of either control peptide or MyD88 inhibitor IMG2005. Twenty-four hours later, a carotid artery catheter was placed and rolling velocity of leukocytes investigated via an *ex vivo* flow chamber system^29^. Leukocytes from C57BL/6 WT mice treated with control peptide rolled at a mean rolling velocity of 0.32±0.01 μm s^−1^ (Fig. 5d). Application of IMG2005 increased rolling velocity to 0.53±0.02 μm s^−1^. Taken together, our results suggest that slow β2 integrin-mediated leukocyte rolling velocity on rmE-selectin/rmICAM-1-coated capillaries is critically dependent on Mrp8/14, TLR4 and MyD88 in C57Bl/6 WT mice.

To address the *in vivo* relevance of our *in vitro* findings in the flow chamber, we used C57BL/6 WT mice and analysed leukocyte rolling velocities in rmTNF-α-stimulated mouse cremaster muscle venules in the presence or absence of Paquinimod. Leukocyte slow rolling depends on E-selectin and the β2 integrins LFA-1 and Mac-1 in this model, as described previously^32^. Mice were pretreated with Paquinimod (10 μg g^−1^ mouse) or carrier substance (control, PBS/10% dimethylsulphoxide (DMSO)) i.p. 1 h before rmTNF-α application. Application of Paquinimod did not affect the number of circulating leukocytes (3,787±420 cells μl^−1^ for PBS/10% DMSO and 3,754±87 cells μl^−1^ for Paquinimod-treated mice, respectively). Leukocytes of control mice rolled with a mean velocity of 3.79±0.34 μm s^−1^ (Fig. 5e). Pretreatment of mice with Paquinimod resulted in a significant increase in rolling velocity compared with control mice with a rolling velocity of 7.50±0.65 μm s^−1^. In a different set of experiments, we pretreated mice via i.p. injection of 30 μg rat anti-mouse TLR4 antibody 1A6 or rat IgG2b isotype control 1 h before rmTNF-α application. Cells from mice treated with rat IgG2b isotype control displayed a mean velocity of 3.41±0.12 μm s^−1^ (Fig. 5f). Pretreatment of mice with rat anti-mouse TLR4 antibody 1A6 resulted in an increase in rolling velocities (7.20±0.39 μm s^−1^), which were similar to levels seen in Paquinimod-treated mice, suggesting that Mrp8/14 and its receptor TLR4 regulate leukocyte rolling velocity *in vivo*.

### Mrp8/14 increases leukocyte adhesion *in vivo*

**Figure 6:**
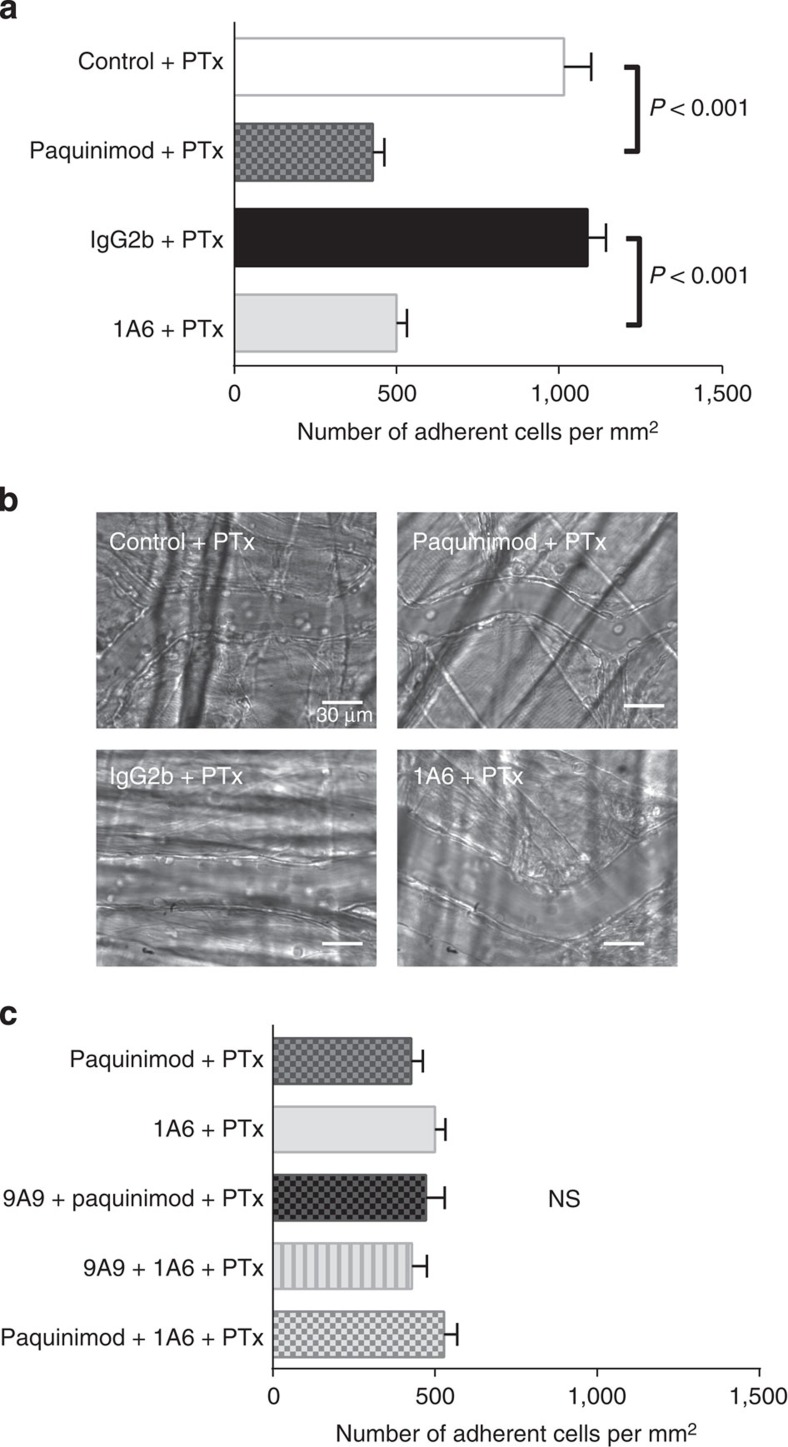
Mrp8/14 increases leukocyte adhesion in TNF-α-stimulated cremaster muscle venules *in vivo.* (**a**) C57BL/6 WT mice were pretreated with carrier substance (PBS/10% DMSO, control)+PTx (white bar), a combination of Paquinimod+PTx (grey spotted bar), rat IgG2b isotype control+PTx (black bar) or a combination of rat anti-mouse TLR4 antibody 1A6+PTx (light grey bar). (**b**) One representative micrograph is shown. (**c**) C57BL/6 WT mice were pretreated with a combination of Paquinimod+PTx (grey spotted bar, same bar as in **a**), a combination of 1A6+PTx (light grey bar, same bar as in **a**), a combination of rat anti-mouse E-selectin Ab 9A9+Paquinimod+PTx (grey spotted bar), a combination of 9A9+1A6+PTx (light grey lined bar) or a combination of 1A6+Paquinimod+PTx (light grey spotted bar). Data are presented as mean±s.e.m. of at least three mice per group, one-way analysis of variance with Tukey's *post-hoc* test.

Finally, we tested the impact of Mrp8/14 on leukocyte adhesion *in vivo*. It was shown previously that in the TNF-α-stimulated inflammation model of the mouse cremaster muscle, blockade of chemokine-induced inside-out signalling by pertussis toxin (PTx) resulted in a markedly reduced number of adherent cells in postcapillary venules of E-selectin ^*−/−*^ (*Sele*^*−/−*^) mice^33^. In contrast, exclusive blocking of Gα_i_-coupled chemokine receptors by PTx or exclusive lack of E-selectin did not significantly alter leukocyte adhesion in TNF-α-stimulated cremaster muscle venules^33^. To demonstrate that Mrp8/14 and TLR4 are involved in E-selectin-triggered leukocyte adhesion, C57BL/6 WT mice were pretreated with Paquinimod+PTx or PBS/10% DMSO (carrier substance, control)+PTx i.p. 1 h before rmTNF-α application. In a second set of experiments, C57BL/6 WT mice were pretreated with rat anti-mouse TLR4 antibody 1A6+PTx or IgG2b +PTx. RmTNF-α was applied to the mouse scrotum and 2 h later mouse cremaster muscle was dissected. Intravascular number of adherent cells per mm^2^ was analysed as described before^34^. The number of adherent cells per mm^2^ was significantly reduced in the presence of Paquinimod and PTx (427±36 cells mm^−2^, Fig. 6a), as compared with control conditions (1,016±84 cells mm^−2^). Consistently, rat IgG2b isotype control+PTx did not influence the number of adherent cells per mm^2^ (1,088±57 cells mm^−2^), whereas the presence of 1A6+PTx reduced the number of adherent cells to 500±32 cells mm^−2^. Representative pictures are shown in Fig. 6b, representative video clips are presented in Supplementary Movies 1 and 2. Application of Paquinimod or 1A6 alone (without PTx) did not significantly reduce the number of adherent cells per mm^2^ as compared with control conditions (DMSO/10% PBS or IgG2b alone; Supplementary Fig. 5).

To exclude potential additive effects of E-selectin-induced adhesion and Mrp8/14-TLR4-induced adhesion, we additionally pretreated mice with a combination of anti-E-selectin antibody 9A9+Paquinimod+PTx or a combination of 9A9+1A6+PTx. Finally, we pretreated mice with a combination of Paquinimod+1A6+PTx (Fig. 6c). None of these combinations further reduced the number of adherent cells per mm^2^ in TNF-α-stimulated mouse cremaster model (472±58, 429±47 and 528±41 cells mm^−2^, respectively). These findings suggest that E-selectin-triggered release of Mrp8/14 interacts with TLR4 to regulate leukocyte adhesion *in vivo*.

## Discussion

**Figure 7:**
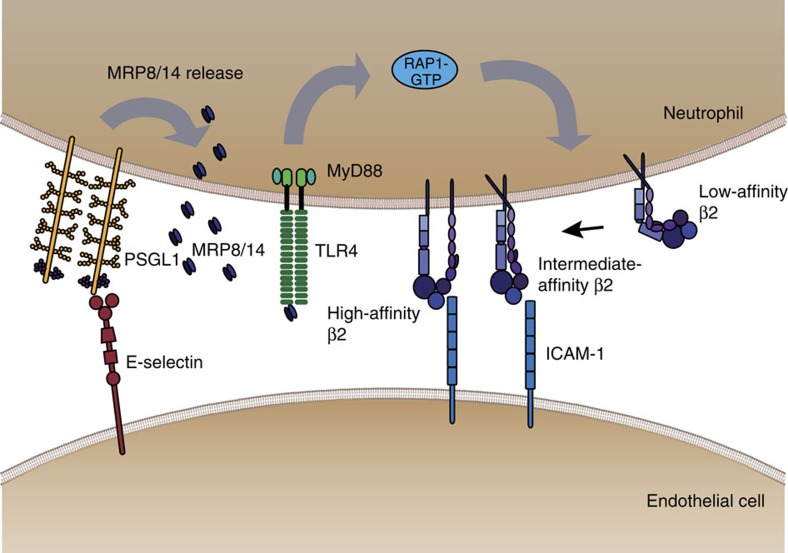
Overview on MRP8/14 and its role in leukocyte recruitment *in vivo.* E-selectin-mediated rolling of leukocytes on inflamed endothelium triggers E-selectin–PSGL-1-dependent release of MRP8/14. Extracellular MRP8/14 in turn binds to TLR4 and through this rapid autocrine extracellular activation loop Rap1-GTP and β2 integrin intermediate and high-affinity activation is induced, which leads to slow rolling and firm leukocyte adhesion on the inflamed endothelium.

Leukocyte recruitment during inflammation follows a well-defined cascade of adhesion and activation events starting with tethering and rolling of leukocytes along the inflamed endothelium^19^. During rolling, integrins expressed on leukocytes undergo conformational changes from a low-affinity bent conformation to an intermediate and finally high-affinity open conformation leading to leukocyte slow rolling and adhesion^35^. Here, we present a new MRP8/14-dependent extracellular activation loop for β2 integrin activation and hence leukocyte recruitment *in vivo* (illustrated in Fig. 7). We show that during E-selectin-dependent leukocyte rolling, the ligation of PSGL1 by E-selectin induces the release of Mrp8/14 complex from neutrophils. Released Mrp8/14 in turn binds to TLR4 expressed on neutrophils, which triggers the activation of Rap1 (Rap1-GTP) leading to intermediate and full-activation of β2 integrins. Using the inhibitor Paquinimod, which blocks binding of Mrp8/14 to its receptor TLR4 (refs 26, 27), we demonstrate that rapidly released Mrp8/14 and neutrophil-expressed TLR4 are a new axis for the regulation of integrin activation thereby affecting integrin-dependent steps of leukocyte recruitment, that is, slow leukocyte rolling and firm leukocyte arrest.

It has been known for several years that activated phagocytes secrete MRP8/14 during inflammatory responses via an alternative pathway bypassing the classical Golgi-route^2^. The secretion was described to be induced during the contact of monocytes with TNF-α-stimulated HUVEC, but not with resting HUVEC^16^. Serum levels of MRP8/14, also known as calprotectin, are strongly elevated during several inflammatory processes^11^,^12^,^13^,^14^,^15^,^16^ and the protein complex is used as an inflammatory biomarker for many years. However, the exact molecules involved in MRP8/14 release and the functional role of serum-circulating MRP8/14 during inflammation remained undefined. Previous studies have suggested that engagement of E-selectin with PSGL-1 during leukocyte rolling on inflamed endothelium triggers an intracellular signalling cascade leading to Rap1-GTP-dependent activation of β2 integrins^21^,^22^,^30^, which results in leukocyte slow rolling (reviewed in ref. 22). Our study indicates that engagement of E-selectin with PSGL-1 does not directly activate Rap1-GTP and β2 integrins, but requires an additional extracellular activation loop involving E-selectin-dependent release of Mrp8/14 with consecutive binding of extracellular Mrp8/14 to neutrophil-expressed TLR4. As PSGL-1 serves also as a ligand for P-selectin^36^, the other selectin expressed on inflamed endothelium, we also tested P-selectin–PSGL-1 interactions^37^ as trigger for Mrp8/14 release. However, we found no significant release of Mrp8/14 via P-selectin, which led us to concentrate on the E-selectin-dependent release of Mrp8/14. Previous intravital microscopy studies demonstrated that E-selectin and CXCR2 cooperate in an overlapping manner to induce firm leukocyte adhesion in TNF-α-stimulated postcapillary venules of the mouse cremaster muscle^33^. Using the same mouse model, our experiments showed that blockade of Gα_i_-coupled signalling through pretreatment of mice with pertussis-toxin only led to a significant drop in leukocyte adhesion, if mice were concomitantly pretreated with TLR4-blocking monoclonal antibody 1A6 or Paquinimod. Additive blockade of E-selectin did not further reduce leukocyte adhesion, underlining our hypothesis that Mrp8/14–TLR4 engagement functions in the same pathway as E-selectin–PSGL1.

Of note, as MRP8/14 is only found in myeloid cells (mostly in neutrophils but also in monocytes), but not in other leukocyte subsets such as B or T lymphocytes, the described E-selectin-triggered and Mrp8/14-dependent mechanism of β2 integrin activation is restricted to the myeloid lineage and might be regarded as a specific activation signal for myeloid cell recruitment. Although mostly neutrophils contributed to the release of Mrp8/14 in our acute *in vivo* models, we found residual Mrp8/14 serum level in mice, where PMN were depleted. This could point to an additional release of Mrp8/14 by circulating monocytes, which have been reported to secrete Mrp8/14 under inflammatory conditions^16^. Furthermore, it was shown previously that Paquinimod inhibits monocyte recruitment to sides of sterile inflammation 20 h after immunization^38^.

Besides identifying an additional extracellular activation loop necessary for β2 integrin activation during engagement of myeloid cells with the inflamed endothelial lining, another remarkable finding of this study consists in the elucidation of TLR4- signalling as a rapid inducer of β2 integrin activation. This has not been described *in vivo* before and expands the function of TLR4 from its role as potent stimulator of nuclear factor-κB to an additional component of a rapid β2 integrin activation pathway triggered through E-selectin-dependent rolling. Signalling via TLR4 is complex, as many different ligands exist, which are able to cause different cellular responses due to the recruitment/activation of different adaptor/signalling molecules downstream of TLR4. In the case of Mrp8/14, ligation of TLR4 by Mrp8/14 induces wide-ranging effects on neutrophils. This includes cytokine and chemokine production, generation of reactive oxygen species^1^ and, as shown here, rapid β2 integrin activation. However, how MRP8/14-stimulated TLR4 signalling pathways induce β2 integrin activation is currently not entirely understood. We identified the involvement of MyD88 and Rap1-GTP downstream of TLR4 stimulation. Interestingly, when we analysed the rolling behaviour of *Tlr4*^*−/−*^ leukocytes, rolling velocities in flow chambers coated with rmE-selectin and rmICAM-1 were equal to those found in C57BL/6 WT mice. Similarly, Andonegui and colleagues reported that leukocyte rolling velocities in TNF-α-treated cremaster muscle venules were similar between WT and *Tlr4*^*−/−*^ mice^37^. However, the same study also reported that in contrast to WT mice, in which local LPS treatment led to a significant drop in leukocyte rolling velocities, leukocyte rolling velocities in *Tlr4*^*−/−*^ mice did not significantly change over baseline following local stimulation with LPS^37^. This implies that TLR4 is involved in regulating leukocyte rolling velocities. However, the involvement of TLR4 does not seem to be constitutive, but rather ligation/stimulation dependent. This could be an explanation for the masking of the leukocyte rolling velocity response in *Tlr4*^*−/−*^ mice. Future studies will be necessary to further clarify this issue and work out the precise signalling pathway of TLR4-dependent β2 integrin activation in neutrophils and monocytes.

MRP8/14 can also bind to other receptors such as the RAGE^39^,^40^ and Paquinimod can also affect the binding of MRP14 to RAGE^26^. Therefore, we also tested a potential involvement of neutrophil-expressed RAGE on Mrp8/14 effector functions. Interestingly, our own Mrp8/14 release studies showed that stimulated Mrp8/14 release led only to an increase in neutrophil surface binding of Mrp8/14 in the presence of TLR4. These results indirectly speak against a role of neutrophil-expressed RAGE as relevant Mrp8/14 ligand in terms of β2 integrin activation. Furthermore, we found that blockade of Mrp8/14 binding to TLR4 with Paquinimod increased *Rage*^*−/−*^ neutrophil rolling velocity to the same extent as seen in C57BL/6 WT mice suggesting no role of neutrophil-expressed RAGE in regulating Mrp8/14-dependent slow leukocyte rolling.

TLR4 and other MRP8/14-binding molecules are also expressed on inflamed endothelial cells and it was shown that MRP8/14 induces a thrombogenic, inflammatory response in HMEC cells *in vitro*^5^. The transcription of proinflammatory chemokines like IL8 and proinflammatory molecules like ICAM-1 was increased upon MRP8/14 treatment of HMEC cells. We show reduced adhesion of leukocytes on inflamed endothelium in C57BL/6 WT mice pretreated with a combination of Paquinimod and PTx or a combination of anti-TLR4 antibody 1A6 and PTx. Although our *in vitro* assays indicate that MRP8/14 exerts its proinflammatory function via binding to TLR4 on neutrophils, MRP8/14 binding to the inflamed endothelium may also contribute to leukocyte recruitment during inflammation *in vivo*.

In summary, our results revise and expand our view on the molecular mechanisms governing leukocyte recruitment during inflammation by introducing a novel MRP8/14-dependent extracellular activation loop as a modulator of the recruitment process *in vivo* (Fig. 7) via bridging the rolling and adhesion processes in an autocrine manner. MRP8/14 released from neutrophils by E-selectin–PSGL1 interactions binds to TLR4, which in turn stimulates a rapid signal transduction cascade leading—via MyD88 and Rap1-GTP—to β2 integrin activation, a necessary step for the induction of slow leukocyte rolling and firm arrest on inflamed endothelium.

## Methods

### Animals

*Cd44*^*−/−*^ mice were provided by Rodger P. McEver (Cardiovacular Biology Research Program, Oklahoma, OK, USA)^41^. *Selpl*^*−/−*^ and *Glg1*^*−/−*^ mice were provided by Dietmar Vestweber (Max Planck Institute for Biochemistry, Münster, Germany)^24^,^36^. *Tlr4*^*−/−*^ mice were obtained from Admar Verschoor (University of Munich, Germany)^42^. *Rage*^*−/−*^ mice were provided by Angelika Bierhaus (University of Heidelberg, Germany, deceased) and Peter Nawroth (University of Heidelberg, Germany)^43^. All mice strains had been backcrossed into C57BL/6 WT background. *Glg1*^*−/−*^ mice are embryonically lethal. Therefore, fetal liver cell chimeras (named *Glg1*^*−/−*^ mice) were generated as described^44^ using *Glg1*-deficient fetal liver cells adoptively transferred into lethally irradiated C57Bl/6 WT mice. C57BL/6 WT mice were obtained from the Janvier Labs (Saint Berthevin, France). All mice were maintained at the Walter Brendel Center for Experimental Medicine, Ludwig Maximilians Universität, Munich, Germany. Eight- to twenty-week-old male mice were used for all experiments. Animal experiments were approved by the government Oberbayern, Germany, AZ 55.2-1-54-2531-134/08, -175/09, -90/09 and -76/12.

### Mrp8/14 release assay

For the *in vitro* release assay, we coated glass dishes overnight with rmE-selectin (CD62E Fc chimera, R&D Systems, 10 μg ml^−1^), rmP-selectin (CD62P Fc chimera, R&D 10 μg ml^−1^), FcR (Miltenyi Biotec GmbH, 10 μg ml^−1^) or PBS at 4 °C. Bone marrow-derived neutrophils were isolated from C57BL/6 WT, *Selpl*^*−/−*^, *Cd44*^*−/−*^ and *Glg1*^*−/−*^ mice using EasySep mouse neutrophil enrichment kit (STEMCELL TECHNOLOGIES) according to the manufacturer's protocol. Purity of isolated cells was over 90%. Glass dishes were transferred into a 24-well plate, blocked with 5% Casein (Sigma-Aldrich) for 2 h and washed with PBS. 5 × 10^5^ neutrophils were reconstituted in HBSS buffer (HBSS containing 1 mM CaCl_2_ und 1 mM MgCl_2_, 10 mM HEPES, 0.25% BSA, 0.1%Glucose, pH7.4) and incubated under shaking conditions on the pre-coated wells at 37 °C for 10 or 30 min. Soluble PMA (Sigma-Aldrich, 1 μM) was used as positive control. Rat anti-mouse E-selectin antibody 9A9 (kind gift from Dr Barry Wolitzky, MitoKor, 10 μg ml^−1^) was used to block E-selectin-induced Mrp8/14 release. Supernatants were collected and analysed by ELISA to determine the concentrations of Mrp8/14 as described earlier^23^.

For the *in vivo* release assay, we injected rmTNF-α (R&D Systems, 500 ng per mouse) into the scrotum of C57BL/6 WT mice, as described earlier^34^. Blocking experiments were performed by i.v. injection of rat anti-mouse antibody 9A9 (30 μg per mouse) 15 min before rmTNF-α application. Neutrophil depletion was induced via i.v. injection of 30 μg rat anti-mouse Ly6G antibody (BioLegend, 1A8) 22 h before rmTNF-α application. 100 μl of mouse blood was collected via retroorbital bleeding before application of anti-mouse E-Selectin antibody or/and rmTNF-α and 2 h after rmTNF-α application. Serum was separated using Microtainer SST Tubes columns (REF 365951, Becton & Dickinson BD) according to the manufacturer's protocol and ELISA was used to determine the concentration of Mrp8/14 as described earlier^23^.

### Autocrine MRP8/14 binding to neutrophils

Bone marrow cells were isolated from C57BL/6 WT mice. 1 × 10^6^ cells were stimulated for 5 min at 37 °C with PBS/0.1% BSA or soluble rmE-Selectin (CD62E Fc chimera, R&D Systems,1 μg ml^−1^). Rat IgG2b isotype control (eBioscience, 10 μg ml^−1^) or rat anti-mouse TLR4 antibody (1A6, NovImmune SA, 10 μg ml^−1^) were co-incubated with tubes containing soluble rmE-selectin. Cells were fixed using BD FACS Lysing solution (BD) and washed with PBS/5% BSA. Thereafter, cells were stained with polyclonal rabbit anti-mouse Mrp14 antibody (1.5 μg ml^−1^, Johannes Roth, University of Münster). Donkey anti-rabbit Alexa Flour 488 (Molecular Probes/Invitrogen, 2.5 μg ml^−1^) was used as secondary antibody. Rat anti-mouse Ly6G pacific blue (1A8, BioLegend, 1 μg ml^−1^) was used to define neutrophils. Amount of receptor-bound Mrp14 on Ly6G^+^/Mrp14^+^ cells was determined using a Beckman Coulter Gallios flow cytometer.

### β2 Integrin activation assay

Human neutrophils were isolated from healthy volunteer blood donors using Polymorphoprep (AXI-SHIELD PoC AS). Enriched neutrophils (5 × 10^5^ sample^−1^) were preincubated with Paquinimod (Active Biotech AB, 10 μg ml^−1^), PBS/10% DMSO (carrier substance), rat anti-human polyclonal TLR4 (PAb-hTLR4, Invivogen, 1 μg ml^−1^) or normal rat IgG Isotype (EMFRET Analytics & Co., 1 μg ml^−1^) for 3 min at 37 °C, respectively. hMRP8/14 was obtained from Johannes Roth, University of Münster. The complex was prepared essentially free of endotoxin contamination from human neutrophils as previously described^45^. hMRP8/14 (5 μg ml^−1^), LPS (Sigma-Aldrich, 1 μg ml^−1^), PMA (Sigma-Aldrich, 1 μM) or PBS/0.1% BSA were added to vials containing mouse anti-human β2 integrin activation antibody KIM127 (Invivo, 10 μg ml^−1^) or mAB 24 (Hycult Biotech, 10 μg ml^−1^) or IgG1 Isotype control antibody (BioLegend, 10 μg ml^−1^) and warmed up to 37 °C for 5 min. Preincubated neutrophils and pre-warmed substance/antibody mix were pooled. Cells were stimulated for another 5 min at 37 °C in a total volume of 40 μl. Reaction was stopped by adding 900 μl ice-cold BD FACS Lysing solution (BD) and tubes were immediately transferred on ice. After 10 min fixation time, cells were washed and stained with goat anti-mouse PE antibody (BD Biosciences Pharmingen, 2.5 μg ml^−1^). To define human neutrophils, cells were finally stained with mouse anti-human CD15-FITC (VIMC6, Miltenyi Biotec GmbH, 1:100) and mouse anti-human CD66abce-APC (TET2, Miltenyi Biotec GmbH, 1:100). Activation status of β2 integrins was determined using a Beckman Coulter Gallios flow cytometer. In a second set of experiments, total Mac-1 and total LFA-1 protein amount was investigated. For this approach, mouse anti-human CD11b (ICRF44, BioLegend, 5 μg ml^−1^) or mouse anti-human CD11a (HI111, BD Biosciences Pharmingen, 5 μg ml^−1^) was used.

### Rap1 activation assay

Human neutrophils were isolated from healthy volunteer blood donors using a double gradient with Histopaque-1119 and 1077 (Sigma-Aldrich). To assess active Rap1 in human neutrophils, a pull-down assay was performed with Rap1 Activation Kit (Milipore) according to the manufacturer's instructions. Briefly, 4 × 10^6^ human neutrophils were incubated or not with E-selectin (1 μg ml^−1^) or hMRP8/14 (5 μg ml^−1^) at 37 °C for 5 min in complete RPMI medium. For inhibitor experiments, cells were pretreated with Paquinimod (10 μg ml^−1^) or rat anti-human TLR4 antibody (1 μg ml^−1^) for 2 min before adding E-selectin or hMRP8/14. Ral GDS-RBD agarose (Milipore, 25 μl) was added to each cell extract and incubated for 50 min at 4 °C. Precipitates were then washed, re-suspended in 30 μl of × 2 sample buffer, boiled for 10 min at 95 °C and separated by 12% SDS–PAGE. Rabbit Rap1 antibody (Milipore, 1 μg ml^−1^) was used to detect active Rap1 (Rap1-GTP). Whole-cell lysates (10 μl) from each cell extract before adding agarose were used to assess total levels of Rap1. The band intensity was quantified by using the Image J software (NIH)^46^. Blood drawing for isolation of human neutrophils from healthy human volunteers was performed with informed consent and approved by the local ethics committee at the University of Dresden, Germany.

### *In vitro* and *ex vivo* flow chamber

To investigate rolling velocities, we used a previously described flow chamber system^29^. Glass capillaries (Rect. Boro Capillaries 0,04 × 0,40 mm ID, VitroCom) were coated overnight with rmE-selectin (CD62E Fc chimera, R&D Systems, 20 μg ml^−1^) or a combination of rmE-Selectin and rmICAM-1 (ICAM-1 Fc chimera, R&D Systems, 15 μg ml^−1^) and blocked with 5% casein (Sigma-Aldrich) for 2 h. Whole blood was collected from C57BL/6 WT, *Rage*^*−/−*^ or *Tlr4*^*−/−*^ mice via the carotid artery and heparinized. Depending on the desired condition, Paquinimod (Active Biotech AB, 10 μg ml^−1^), rat IgG2b (BD Biosciences Pharmingen, 10 μg ml^−1^), rat anti-mouse TLR4 antibody 1A6 (1A6, NovImmune SA, 10 μg ml^−1^) or Lovastatin (Calbiochem, Merck KGaA, 100 μM) was added to the blood sample, respectively. Samples were preincubated for 3 min at 35 °C and perfused through the microflow chambers using a high precision perfusion pump at a shear stress level of 2.7 dyn cm^−2^. For *ex vivo* flow chamber assays, C57BL6/WT mice were pretreated by i.p. application of either control peptide (BIOMOL, 1 mg kg^−1^) or MyD88 inhibitor IMG2005 (BIOMOL, 1 mg kg^−1^) for 24 h. Carotid artery catheter was placed and mouse blood directly perfused through the microflow chamber at a shear stress level of 3–4 dyn cm^−2^. One representative field was recorded for 5 min using an Olympus BX51WI microscope with a CCD camera (model CF8/1, Kappa) and a water immersion objective (x40/0.8 NA, Olympus). Rolling velocity of the cells was determined using Fiji software^46^.

### TNF-α-induced inflammation model of mouse cremaster muscle

The TNF-α-induced inflammation model of the mouse cremaster muscle was performed as described previously in C57BL/6 WT mice^34^. Briefly, C57BL/6 WT mice were pretreated i.p. with a combination of Paquinimod (Active Biotech AB, 10 mg kg^−1^)+PTx (Sigma-Aldrich, 4 μg per mouse) or rat anti-mouse TLR4 antibody (1A6, NovImmune SA, 100 μg per mouse)+PTx (Sigma-Aldrich, 4 μg per mouse). PBS/10%DMSO (carrier substance)+PTx and rat IgG2b (eBioscience, 100 μg per mouse)+PTx were used as controls, respectively. In a second set of experiments, rat anti-mouse anti-E-selectin antibody 9A9 (100 μg per mouse) was applied concomitantly. Depending on the desired condition and for the investigation of *in vivo* rolling velocities, Paquinimod, PBS/10% DMSO, 1A6 and rat IgG2b isotype control were applied without PTx. 500 ng of rmTNF-α (R&D Systems, 500 ng mouse^−1^) was applied to the mouse scrotum 1 h later. After 2 h of rmTNF-α application, cremaster muscle was dissected. Mean rolling velocities and number of adherent cells per mm were determined using intravital microscopy (Olympus BX51WI microscope, water immersion objective x20, 0.95 numerical aperture, Olympus). All scenes were recorded using a CCD camera (model CF8/1, Kappa) and virtual dub software for later off-line analysis. During the entire observation, the cremaster muscle was superfused with thermo-controlled (35 °C) bicarbonate-buffered saline. Postcapillary venules under observation ranged from 20 to 40 μm in diameter. Microvascular parameters (venular diameter, venular vessel segment length) were determined using Fiji software^46^.

### Statistical analyses

All data were analysed and plotted using Graph Pad Prism 6.05 Software (GraphPad Software Inc.). For pairwise comparison of experimental groups, a paired *t*-test was performed. Depending on the condition, we used one-way analysis of variance with either Dunnett's *post-hoc* test (comparison of experimental groups against control) or Tukey's *post-hoc* test (comparison of all experimental groups against each other) or a two-way analysis of variance with Tukey's *post-hoc* test (comparison of paired experimental groups against each other) for multiple comparison. *P*-values<0.05 were considered statistically significant.

## Author contributions

M.P. designed, performed and analysed the experiments and wrote the manuscript; A.R., M.K., K.J.C, X.C-E., S. Bieber, S. Bierschenk, I.R., K.H., M.M. and S.G. performed and analysed experiments; C.F.N., T.K.E and R.I. analysed experiments; U.K., R.P.M., D.V., A.V. and T.L. provided reagents critical for the project; T.C. and J.R. contributed to the design of the experiments and the interpretation of the data; and revised the manuscript. T.V. and M.S. designed the experiments, interpreted the data and wrote the manuscript.

## Additional information

**How to cite this article:** Pruenster, M. *et al.* Extracellular MRP8/14 is a regulator of β2 integrin-dependent neutrophil slow rolling and adhesion. *Nat. Commun.* 6:6915 doi: 10.1038/ncomms7915 (2015).

## Supplementary Material

Supplementary Figures Supplementary Figures 1-5

Supplementary Movie 1 Leukocyte adhesion efficiency in vivo. C57BL/6 WT mice were pretreated with rat IgG2b Isotype control + PTx. RmTNF-α was applied to the mouse scrotum. The scene was recorded for 2 min using a CCD camera (model CF8/1, Kappa). Movie was initially generated as uncompressed .avi file and converted into .m4v format (25frames/sec) using VLC media player software.

Supplementary Movie 2 Leukocyte adhesion efficiency in vivo after blocking TLR4. C57BL/6 WT mice were pretreated with rat anti-mouse TLR4 antibody 1A6 + PTx. RmTNF-α was applied to the mouse scrotum. As in Supplementary movie S1, the scene was recorded for 2 min, and movie generated as uncompressed .avi files and then converted into .m4v format (25frames/sec).
