# Supplementary material for: Extracellular MRP8/14 is a regulator of β2 integrin-dependent neutrophil slow rolling and adhesion
Source: Nat Commun. 2015 Apr 20;6:6915. doi: 10.1038/ncomms7915 (PMC4411303; doi:10.1038/ncomms7915)
Supplement: Supplementary Figures — 1-5 [file ncomms7915-s1.pdf]

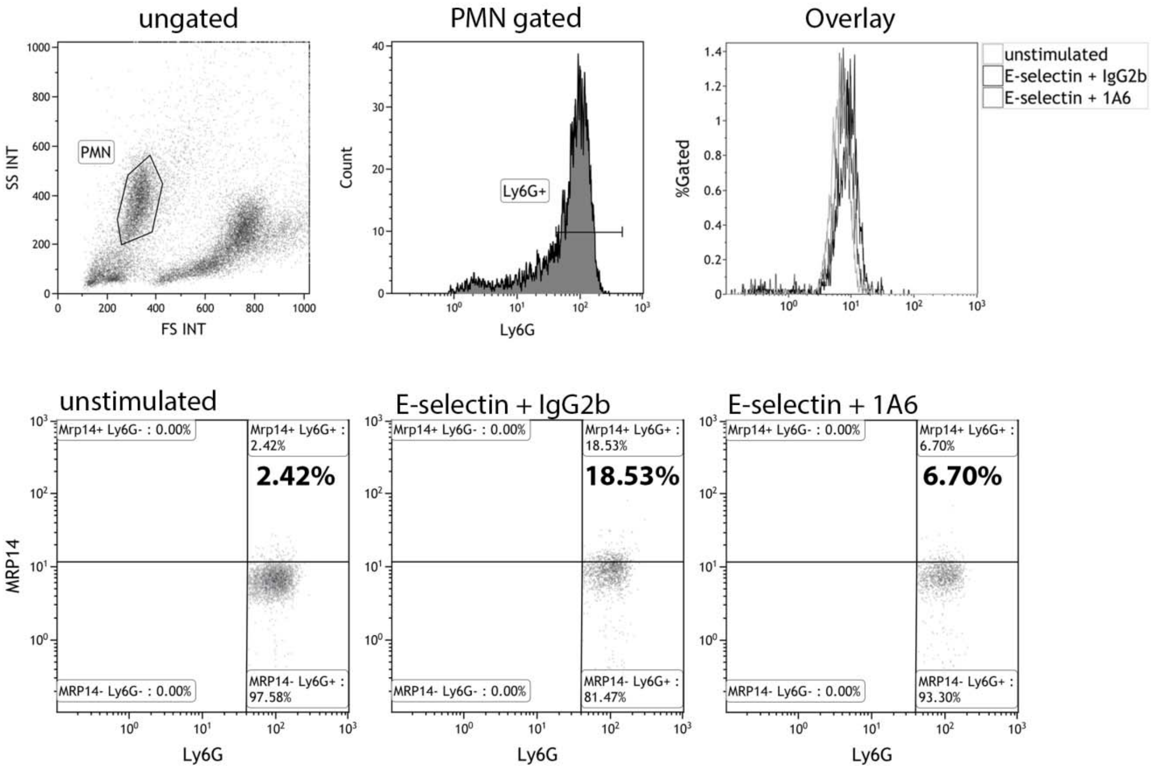

Supplementary Figure 1 Released Mrp8/14 binds to TLR4 on leukocytes in an autocrine fashion – gating strategy. Amount of receptor bound Mrp14 on Ly6G+/Mrp14+ cells was determined using a Beckman Coulter Gallios^{TM} flow cytometer. One representative plot out of 4 is shown.

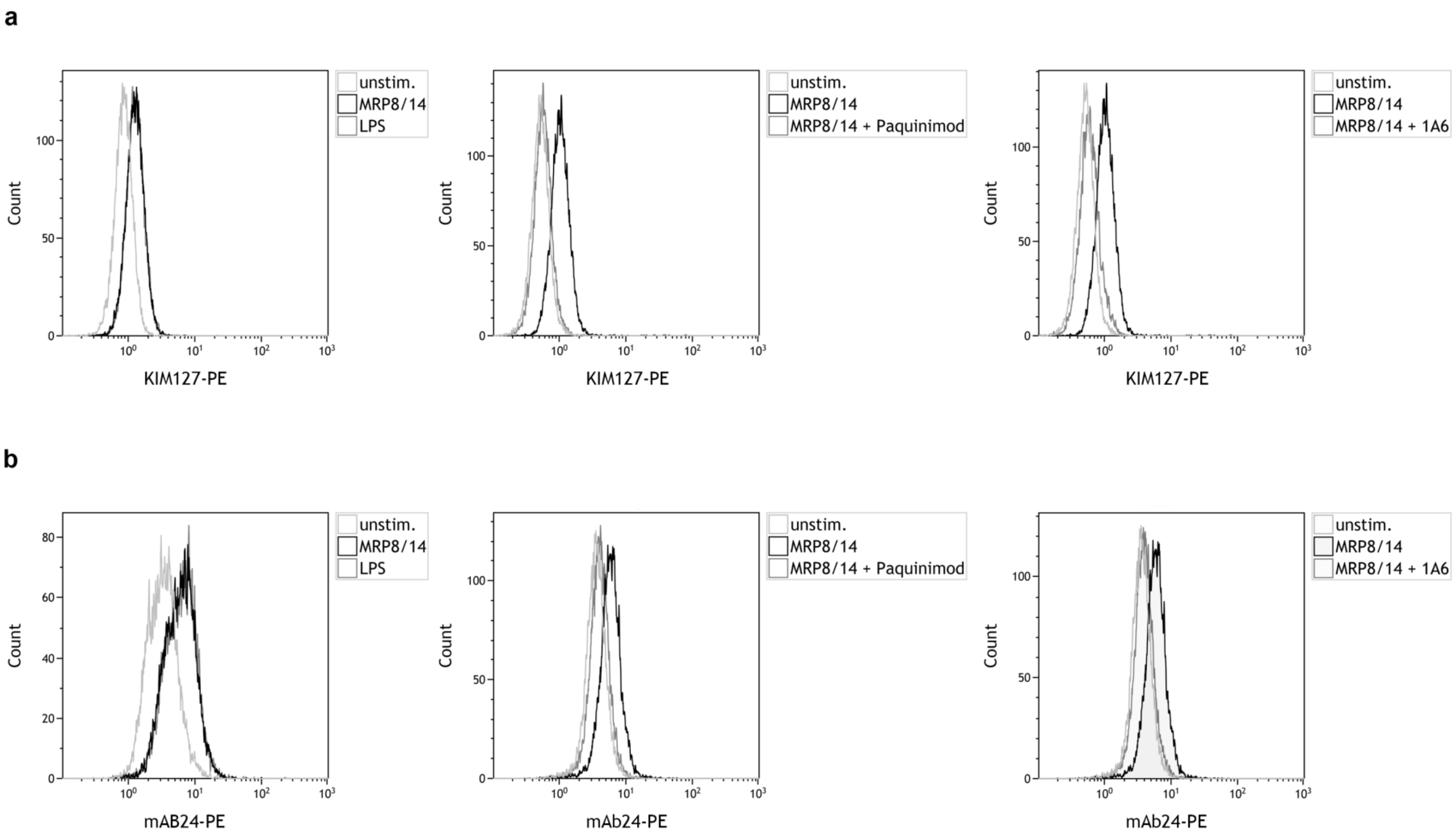

Supplementary Figure 2 hMrp8/14 activates β2 integrins via TLR4 – representative Histogramm plots. Amount of receptor bound KIM127 **(a)** and mAB24 **(b)** on CD15+/CD66abce+ cells was determined using a Beckman Coulter Gallios^{TM} flow cytometer. Gates were set by using an isotype control.One representative plot out of ≥ 3 is shown.

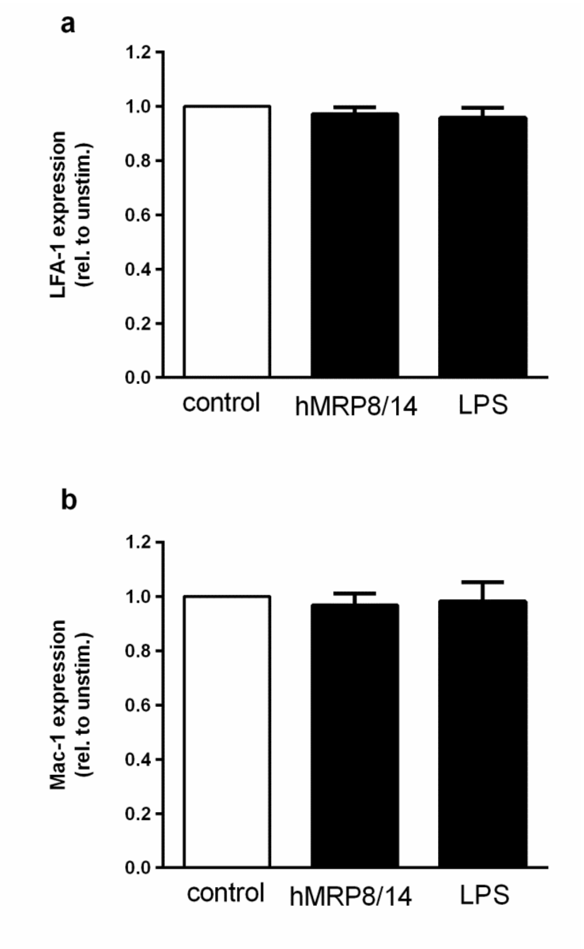

Supplementary Figure 3 Influence of hMRP8/14 and LPS on total surface expression of LFA-1 and Mac-1. Neutrophils from healthy blood donors were stimulated for 5 min at 37°C with or without hMRP8/14 or LPS. Cells stimulated without further substances were termed unstimulated control cells. Values of LFA-1 and Mac-1 binding from unstimulated controls were set to 1. Values from stimulated cells were then calculated as ratio to unstimulated. Surface expression levels are given of total LFA-1 in **(a)** and for total Mac-1 in **(b)**. Gates were set by using an isotype control. Data are presented as mean ± SEM, one way ANOVA with Tukey's post-hoc test.

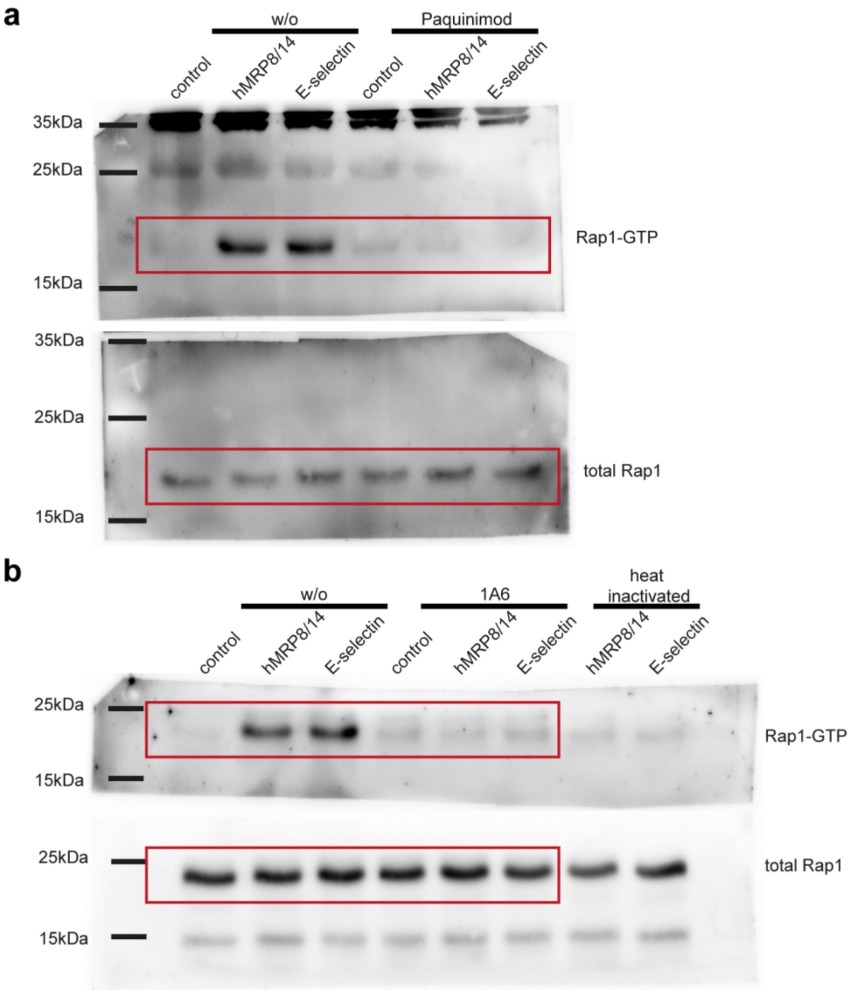

Supplementary Figure 4 MRP8/14 activates Rap1 via TLR4 - Gel documentation. Full immunoblots with indicated areas of selection.

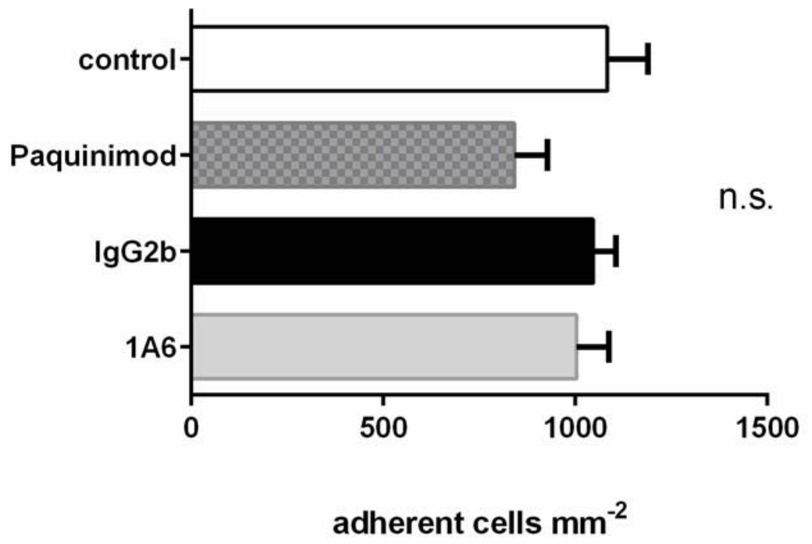

Supplementary Figure 5 Paquinimod and 1A6 alone do not alter number of adherent cells mm^{-2}. C57BL/6 WT mice were pretreated with carrier substance (PBS/10%DMSO, control, white bar), Paquinimod (grey spotted bar), rat IgG2b isotype control (black bar) or a combination of rat anti-mouse TLR4 antibody 1A6 (light grey bar). RmTNF-α was applied to the mouse scrotum and intravascular number of adherent cells mm^{-2} was analyzed. Data are presented as mean ± SEM of at least 3 mice per group, one way ANOVA with Tukey's post-hoc test.
